# Detection of Frailty in Primary Care

**DOI:** 10.1002/agm2.70050

**Published:** 2025-10-10

**Authors:** Duncan Forsyth

**Affiliations:** ^1^ School of Healthcare & Medical Sciences Sunway University, Malaysia and Retired Consultant Geriatrician From Cambridge University Hospitals NHS Foundation Trust, Addenbrooke's Hospital Kuala Lumpur UK

**Keywords:** detection, frailty, primary care

As populations age, while those aged 65 years can expect to live on average around half of their remaining life expectancy in good health, the likelihood of their being disabled and/or experiencing multiple chronic and complex health conditions increases with advancing age. Thus, as life expectancy increases, so does the amount of time spent in poor health, which means the number of people living with complex and multiple conditions will also increase [1]. Those living in deprived areas or experiencing poverty throughout life are more likely to develop frailty earlier than those from wealthier backgrounds [2].

Although the risk of becoming frail increases with advancing age, frailty is not an inevitable consequence of aging, nor is it confined to the older population [3]. Rockwood [4] defined frailty as “a failure to integrate responses in the face of stress. This is why diseases manifest themselves as the “geriatric giants”….functions …such as staying upright, maintaining balance and walking are more likely to fail, resulting in falls, immobility or delirium”; in other words, frailty equates to poor functional and often cognitive reserve and is more related to biological than chronological age. The “geriatric giants,” also known as the 4 “I's: falls (instability), confusion (intellectual failure), immobility, and incontinence; to which might be added iatrogenic disease; may present as any combination of falls, confusion, immobility, and incontinence.” Particularly good markers of frailty are falls and delirium. Frailty is common, affecting approximately 10% of those aged over 65 years and 25%–50% of those aged over 85 years [5] and leads to a higher risk of sudden deterioration in physical and mental health, often precipitated by a relatively minor event [5] with a higher risk of mortality [6]; and is distinct from living with one or more long‐term conditions. Around 47% of hospital inpatients over 65 are affected by frailty [7]; clinical frailty score [8] correlates with increased mortality rates, with a 25% increase in mortality risk for each point increase in clinical frailty score (CFS) [9], and with increased length of hospital stay [10].

So, as there is a risk of significant harm to those who are frail (those with complex needs), if healthcare systems and interventions are planned without consideration of their frailty, the purpose of screening for frailty is to identify: those at risk; what their risks are; and to implement what is necessary to minimize these risks, in order to reduce the risk of harm. That is, to do more good than harm! As complex frail older people have become major consumers of healthcare, there is a need for healthcare systems to move from single‐condition disease‐oriented care to individualized goal‐oriented coordinated care and support; doing so should benefit both consumers and the health and care systems they use [11, 12, 13, 14]. The majority of the population is low‐risk patients, with no or only minor conditions that are easily managed in primary care; up to 35% are rising‐risk patients, with one or more chronic conditions, of whom around 18% each year will become high‐risk patients; the frailest cohort with multiple complex conditions and comorbidities accounts for around 5% of the population who are at the highest risk of medical crises and are the highest‐cost patients [15]. If we can anticipate and delay the onset of poor health, to minimize the consequences of multiple conditions, there should be a consequential reduction in the need for multiple, unplanned and/or urgent interactions with the health system [11, 16, 17, 18] and improvement in people's experience [19, 20].

Routine frailty identification using health record data, and direct patient assessment to identify those at greater risk of adverse outcomes was embedded into the UK General Practice contract in 2017–2018. Screening may be opportunistic, by assessing for frailty in people who present to health and care services; or population‐based, where a more systematic approach is taken to proactively identify people who might be living with frailty. Physical frailty indicators are predictors of impairment in activities of daily living (ADL) in those aged over 65 years living in the community. Monitoring these indicators may be useful for identifying elderly people who could benefit from an intervention to prevent ADL disability. Slow gait speed and low physical activity/exercise are the most powerful predictors, followed by weight loss, reduced lower extremity function, poor balance, and low muscle strength [21]. A multi‐disciplinary approach should be used to identify those aged > 65 years living with frailty, and the degree of their condition (mild, moderate, severe); a dedicated individual from the primary care team should coordinate frailty care for those living with frailty, by selecting the most appropriate care to meet the principal needs of the person living with frailty. This process requires a holistic and comprehensive assessment of need, care and support planning to promote self‐management where feasible, community care and support to address issues such as social isolation and loneliness, and social care and support to meet care needs [22]. Comprehensive Geriatric Assessment (CGA) is the Gold Standard for assessing those living with moderate–severe frailty and leads to better outcomes of reduced readmissions; reduced institutionalization; lower healthcare costs; and a 30% higher chance of being alive and at home at 6 months [23, 24].

The tools used for screening for frailty will depend upon availability and user familiarity. In this author's opinion: “it matters not what tool you use but that you use one with which you are familiar/comfortable using and that you understand its functionality and limitations.” In the UK, the Electronic Frailty Index (eFI) uses routinely collected data coded within the general practice electronic patient records to calculate a frailty index; this then helps to identify those patients who would benefit from further assessment [25]. Higher eFI scores indicate increasing frailty and greater risk of adverse outcomes, for example, those with an eFI score > 0.36 have a sixfold increased risk of admission to a care home in the next 12 months and a fivefold increase in mortality risk, compared to fit older people. However, not all practices have access to the eFI, but those that do can use this information to stratify their population aged 65 years and over by degree of frailty (not frail; mild, moderate, or severe frailty). The frailty diagnosis, using eFI, should be verified (systematically or opportunistically) for those in the moderate and severe groups by direct assessment using the CFS or a similar validated tool, for example, Edmonton Frailty Scale [26]; mild frailty equates to a CFS score of 4–5, moderate frailty to a CFS score of 6, and severe frailty to a CFS score of 7 or above.

What if you don't have access to an electronic frailty index; how might you identify those most likely to be frail? Start by identifying those groups where frailty is most likely to be prevalent:

- Care home residents.
- Those known to adult social care and support services with continuous support needs.
- Those housebound or known to community nurses.
- People known to be living with dementia.
- Those aged over 65 years. who have experienced one of the major frailty syndromes, that is, recurrent falls, altered mobility (e.g., sudden change in mobility, “gone off legs,” “stuck in the toilet”), delirium, a change in continence (new onset or worsening of urine or fecal incontinence).
- Those most susceptible to side effects of medication (those aged over 65 years. with multimorbidity due to 4 or more long term conditions, or those on > 5 medications).
- Those with complex neurological conditions, for example, stroke, multiple sclerosis, Parkinson's.
- Those on end of life (EOL) register or cancer care lists.
- Everyone aged over 85 years.

What simple screening tools might you use? [27].

- Walking speed < 0.8 m/s.
- Timed Up and Go test (TUG) > 10 s (the time taken to stand up from a chair, walk 3 m, turn, walk back to the chair and sit down).
- PRISMA 7 questionnaire score > 3 (good sensitivity, moderate specificity, so high false +ve rates).

Once identified, the two key elements of assessing those living with frailty are: falls risk identification and the next steps to reduce this risk, which should follow National Institute for Health and Care Excellence (NICE) guideline 161 [28]; and an annual medication review, possibly using STOPP START criteria [29]. For patients with frailty and multimorbidity (2 or more long‐term conditions), NICE guideline 56 provides guidance on tailoring care for people with multimorbidity [30].

Frail older people are becoming, and in many countries already are, the “core business” of acute hospitals, making Geriatrics “too important to be left to Geriatricians” [31]. Whether working in primary or secondary care, all clinicians will need to identify those with frailty or at risk of becoming frail, in order to minimize the burden falling upon the individual, their families, the health and social care systems, and the economy. Past models of health care systems designed for people with single pathology are no longer fit for purpose when the major users now have multiple pathology with complex care needs. In conclusion, there are several key areas for focus:

- Ensure system wide recognition of the signs of frailty.
- Know what to do when signs of frailty are found.
- Use a standardized way of stratifying frailty.
- Ensure a means for identifying frailty and frailty risk (consider a “vulnerable elders register”).
- Provide support for people living with mild frailty and encourage people to age well; include them in their care.
- Provide support for people living with moderate frailty (CGA, home and community rehabilitation, surveillance for deterioration).
- Provide support for people living with severe frailty (carer support/education; care home support; end of life care and advanced care planning; admissions avoidance strategies).
- Tailor evidence‐based medicine (EBM) to the individual (as complex frail older people are rarely included in the research that underpins EBM).
- Use a patient‐centered approach to support and services (assess using patient‐reported outcome measures {PROM} and patient‐reported experience measures {PREM}) to generate a personalized shared‐care support plan.

Finally, frailty should be considered as a progressive, long‐term condition that can be ameliorated but not cured, and so requires an individualized treatment plan, with coordinated, person‐centered proactive care that should involve family carers [32]; it is costly at the individual and societal level; individuals are prone to episodic crises and so management tries to avoid/minimize these, thereby helping to minimize any adverse impact on life experience. It is important to recognize the uncertainty in prognosis and to be candid with the patient and family regarding the uncertainty in prognosis [33].
